# Targeting Virulence in *Staphylococcus aureus* by Chemical Inhibition of the Accessory Gene Regulator System *In Vivo*

**DOI:** 10.1128/mSphere.00500-17

**Published:** 2018-01-17

**Authors:** Akram M. Salam, Cassandra L. Quave

**Affiliations:** a Program in Molecular and Systems Pharmacology, Laney Graduate School, Emory University, Atlanta, Georgia, USA; b Center for the Study of Human Health, Emory University, Atlanta, Georgia, USA; c Department of Dermatology, Emory University School of Medicine, Atlanta, Georgia, USA; d Antibiotic Resistance Center, Emory University, Atlanta, Georgia, USA; University of Rochester

**Keywords:** *Staphylococcus aureus*, antimicrobial activity, antimicrobial agents, quorum sensing, virulence

## Abstract

Methicillin-resistant *Staphylococcus aureus* (MRSA) presents one of the most serious health concerns worldwide. The WHO labeled it as a “high-priority” pathogen in 2017, also citing the more recently emerged vancomycin-intermediate and -resistant strains. With the spread of antibiotic resistance due in large part to the selective pressure exerted by conventional antibiotics, the use of antivirulence strategies has been recurrently proposed as a promising therapeutic approach. In MRSA, virulence is chiefly controlled by quorum sensing (QS); inhibitors of QS are called quorum quenchers (QQ). In *S. aureus*, the majority of QS components are coded for by the accessory gene regulator (Agr) system. Although much work has been done to develop QQs against MRSA, only a few studies have progressed to *in vivo* models. Those studies include both prophylactic and curative models of infection as well as combination treatments with antibiotic. For most, high efficacy is seen at attenuating MRSA virulence and pathogenicity, with some studies showing effects such as synergy with antibiotics and antibiotic resensitization. This minireview aims to summarize and derive conclusions from the literature on the *in vivo* efficacy of QQ agents in MRSA infection models. *In vitro* data are also summarized to provide sufficient background on the hits discussed. On the whole, the reported *in vivo* effects of the reviewed QQs against MRSA represent positive progress at this early stage in drug development. Follow-up studies that thoroughly examine *in vitro* and *in vivo* activity are needed to propel the field forward and set the stage for lead optimization.

## Abstract (precis)

Methicillin-resistant *Staphylococcus aureus* (MRSA) presents one of the most serious health concerns worldwide. The WHO labeled it as a “high-priority” pathogen in 2017, also citing the more recently emerged vancomycin-intermediate and -resistant strains.

## INTRODUCTION

The alarming rise of antibiotic resistance presents significant challenges to human health on a global scale (1). Methicillin-resistant *Staphylococcus aureus* (MRSA), particularly community-associated (CA-MRSA) strains such as USA300, presents a unique threat due to its hypervirulent nature (2). While *S. aureus* causes an array of diseases such as infective endocarditis, osteoarticular infections, prosthetic device infections, bacteremia, and pneumonia, approximately 90% of *S. aureus* infections are skin and soft tissue infections (SSTIs) (3, 4). For the treatment of MRSA infections, physicians are now turning more than ever to antibiotics of last resort. What is more, resistance to even these antibiotics, like linezolid, has begun spreading (5–7). A major contributor to this phenomenon is that conventional antibiotics target cellular processes necessary for bacterial survival (8). As such, great selective pressure is exerted on bacterial populations, which divide and mutate rapidly, to genetically develop resistance (9, 10). A different type of target in *S. aureus*, namely, quorum sensing (QS), shows promise for circumventing this shortcoming (11, 12).

QS is a system by which many bacterial species monitor their local population density via secretion and detection of small autoinducer molecules in order to modulate gene expression (13, 14). All QS systems possess three common mechanisms: autoinducer production, accumulation, and detection. The three most thoroughly studied QS systems are based on the following autoinducers: the acylated homoserine lactones utilized by Gram-negative bacteria (also called autoinducer-1), the peptide signals used by Gram-positive bacteria, and autoinducer-2, which is utilized by both (15). Additional QS signaling molecules include autoinducer-3, the *Pseudomonas* quinolone signal (PQS), and the diffusible signal factor (DSF). QS has been established as a mediator of virulence through which bacteria regulate genes involved in host invasion, immune evasion, and dissemination. Interest in quorum sensing inhibition as an antivirulence strategy against a variety of human pathogens has increased greatly over the past decade (16). In the case of *S. aureus*, the field continues to expand.

## THE Agr SYSTEM AS AN EMERGING TARGET FOR DRUG DEVELOPMENT IN VIRULENT *S. AUREUS* INFECTION

**FIG 1:**
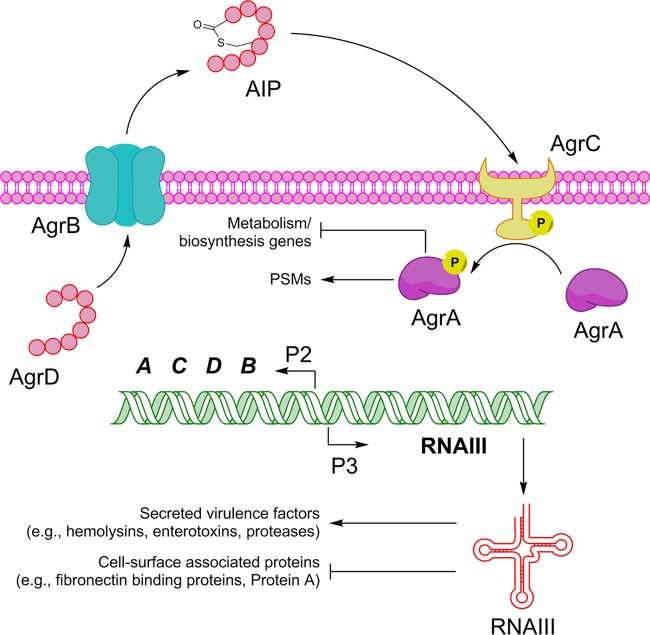
Schematic of the *S. aureus* accessory gene regulatory (Agr) system.

In *S. aureus*, the majority of QS components are encoded by the accessory gene regulator (Agr) system, which regulates most temporally expressed *S. aureus* virulence factors (Fig. 1) (14, 17). When the concentration of *S. aureus*’s secreted autoinducer peptide (AIP) reaches a critical threshold value as the result of increased bacterial population density, the majority of virulence response systems are triggered. This includes production of virulence factors and mechanisms of antibiotic resistance (e.g., upregulated efflux pump) (18–23). The *agr* chromosomal locus encodes two transcripts, RNAII and RNAIII, which are divergently transcribed from the P2 and P3 promoters, respectively (24). The RNAII segment of *agr* is an operon of four genes, *agrBDCA*, which encode all the main components of QS (14, 17). AgrB is an integral membrane endopeptidase that converts the precursor AIP, AgrD, to mature AIP and exports it (17, 25). AIP is recognized by the membrane-bound receptor histidine kinase AgrC, which subsequently phosphorylates AgrA in the cytosol (26). AgrA is a member of a family of conserved response regulators with CheY-like receiver domains; upon phosphorylation, it binds to P2 and P3, upregulating *agr* transcription of RNAII and -III (27). Additionally, AgrA directly induces expression of several phenol-soluble modulins (PSMs). The RNAIII segment of *agr* codes for a regulatory, small RNA molecule that acts as the primary effector of the quorum sensing system by upregulating virulence factor expression and downregulating cell surface protein expression (28, 29). The RNAIII transcript also contains the 26-amino-acid delta-toxin gene (*hld*). Four allelic groups of *agr* have been identified within *S. aureus*, categorized as *agr* I to IV (30).

Inhibiting QS would constrain *S. aureus*’s ability to kill host cells, evade host immunity, and disseminate. Indeed, it is through the production of QS-regulated toxins such as delta-toxin, Panton-Valentine leukocidin, and staphylococcal enterotoxin C that the pathogen achieves these feats (31–35). Additionally, targeting *S. aureus* QS, and thus virulence, rather than survival has been proposed to exert less selective pressure for the development of resistance than conventional antibiotics (12, 36, 37). This may indeed be the case, with numerous QS inhibitors, or quorum quenchers (QQs), demonstrating a markedly diminished ability to give rise to resistance relative to conventional antibiotics in preliminary studies (38, 39). Moved by this promising strategy, much research has been devoted to QQ discovery against MRSA, yielding a wealth of *in vitro* data and an increasing number of QQ agents tested *in vivo*. Here, we review the current status of the field by examining reports on *in vivo* testing of QQs as an antivirulence strategy against MRSA.

## SYNTHETIC QUORUM QUENCHERS

The synthetic quorum quenchers discussed here are small molecules and antisense nucleic acids. All were discovered via various approaches, including combinatorial chemistry, high-throughput screening, and software prediction.

### Biaryl hydroxyketones.

**FIG 2:**
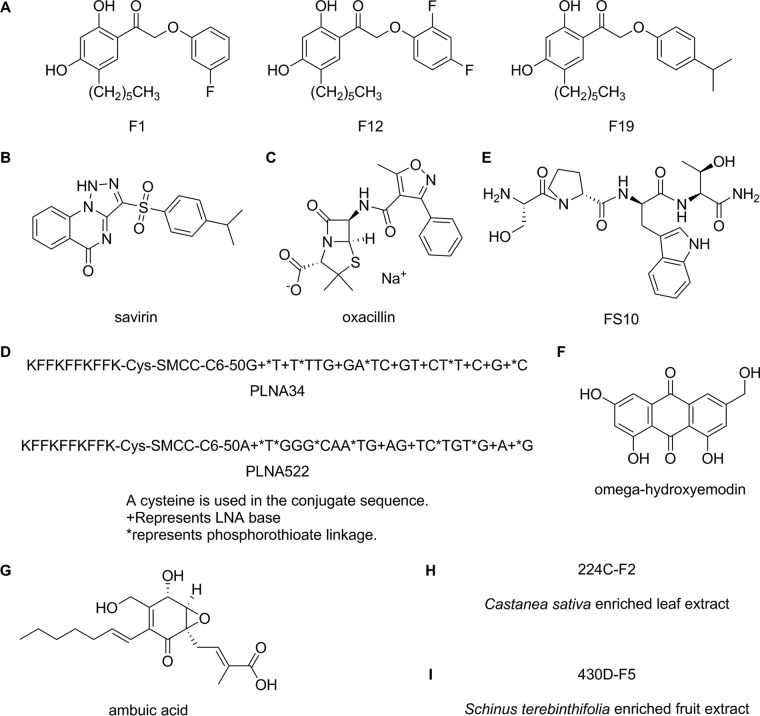
Quorum-quenching compounds tested *in vivo* for inhibition of MRSA virulence. (A) Biaryl hydroxyketones F1, F12, and F19. (B) Savirin. (C) Oxacillin. (D) Peptide-conjugated locked nucleic acids. (E) Tetrapeptide RIP derivative FS10. (F) ω-Hydroxyemodin. (G) Ambuic acid. (H) Enriched *Castanea sativa* leaf extract 224C-F2. (I) Enriched *Schinus terebinthifolia* fruit extract 430D-F5.

Biaryl hydroxyketones have been identified that target the response regulator AgrA by disrupting the AgrA-P3 interaction and, consequently, virulence factor production (40). In a follow-up study involving a combinatorial library synthesized based on the most efficacious biaryl hydroxyketone, compound F12 demonstrated 98% inhibition of rabbit erythrocyte hemolysis *in vitro* by MRSA (USA300 strain) at 1 µg/ml (41). In their latest work, Kuo et al. examined the *in vivo* efficacy of F12 as well as F1 and F19 (Fig. 2A), two other compounds that displayed similarly high bioactivity (42). They utilized a murine wound infection model and a *Galleria mellonella* insect larva model of infection. The infecting strain used in both cases was a USA300 clinical isolate from a patient from Metro Health Medical Center, Cleveland, OH.

The three compounds were first shown to exhibit no toxicity up to 200 µM in a lactate dehydrogenase (LDH) assay of the murine macrophage cell line J774.2. In the murine wound infection model, a dorsal excisional wound created with a punch biopsy tool was inoculated with 1 × 10^7^ CFU of the USA300 strain. Topical application of F12 twice a day at 20 mg/kg of body weight for 8 days performed comparably to F1 and promoted modest wound healing relative to vehicle throughout the treatment duration. In uninfected wounds, treatment offered no healing advantage. Bacterial burdens, assessed 1 day after administration of the final treatment dose, were found to be statistically similar in all treatment groups.

In a *Galleria mellonella* larva model of MRSA infection, 2 × 10^7^ CFU of a USA300 strain was administered to establish infection along with a 20-mg/kg dose of F1, F12, or F19 as treatment. The treatment was repeated every 6 h over the course of up to 84 h. Administration of F12 increased survival from 12 h in untreated controls to 42 h, being outperformed by F1 (60 h) and F19 (66 h). Combination therapy of each of the biaryl hydroxyketones with the β-lactam antibiotic cephalothin or nafcillin, to both of which USA300 is resistant in monotherapy, further increased survival. This result indicates a synergism between the QQ compounds and the antibiotics. F19 was found to offer the greatest survival advantage, up to 84 h, with each antibiotic. Finally, combination with 1 µg/ml F1, F12, or F19 reduced the MIC of nafcillin and cephalothin from 60 and 40 µg/ml to 1 µg/ml, the level of current MRSA antibiotic treatments.

### Savirin.

Savirin is a small molecule with QQ activity identified via high-throughput screening by Sully et al. (Fig. 2B) (38). The screen tested for inhibition of fluorescence in an *S. aureus* reporter strain of AIP-induced *agr*::P3 activation. Savirin, in turn, also exhibited minimal effects on exponential-phase growth in dose-response experiments. Sully et al. showed that savirin inhibited *agr*::P3 activation in all *agr* allelic types of *S. aureus* and that it also inhibited RNAIII production and *agr*-dependent stationary-phase growth. Favorably, the latter effects were not exerted in *Staphylococcus epidermidis*, an important skin commensal in host defense (43). Due to this selectivity over *S. epidermidis*, AgrA was pursued as savirin’s likely molecular target rather than AgrC, where the two residues that are critical for *agr* activation are conserved between the species (44). AgrA_C_ (AgrA C-terminal DNA-binding domain) was confirmed as the target via *in silico* docking, electrophoretic mobility shift assay, and an *agr*::P3 *lux* reporter strain with constitutively produced AgrA. They then showed that treatment with savirin resulted in inhibition of *agr*-specific transcription regulation of major SSTI virulence factors. This was shown via microarray analysis, quantitative real-time PCR (qRT-PCR), and direct measurements of virulence factor function.

The group studied an air pouch skin infection model using Nox2^−/−^ mice, which allowed for maximal *S. aureus agr* expression. A load of 4 × 10^7^ CFU of the USA300 *agr* type I MRSA strain (Los Angeles Clone [LAC]) expressing a fluorescent reporter of *agr*::P3 activation (AH1677) was subcutaneously coadministered to the mice with 10 µg savirin (45). Savirin treatment led to a marked reduction in *agr*::P3 activation in bacteria. Additionally, 24 h after infection, weight loss due to infection (a measure of morbidity) was greatly attenuated, as was bacterial burden in the pouch and in the systemic circulation. In mice infected with LAC Δ*agr*, 10 µg savirin had no effect on weight loss or bacterial burden, indicating that savirin selectively inhibits *agr* activation.

The group also evaluated savirin in a model of *agr*-dependent dermonecrotic skin infection in SKH1 hairless immunocompetent mice (46, 47). A bacterial load of 4 × 10^7^ CFU of LAC was coadministered with 5 µg savirin. Throughout the course of the 7-day treatment, abscess and ulcer areas were severely diminished relative to vehicle control. On day 3, bacterial loads in the treated and control mice were the same, indicating that savirin treatment did not affect bacterial viability at this time point. On day 7, a decrease in bacterial population of approximately 2 log units was observed in both the abscesses and systemic circulation (as observed from the spleen). This was attributed, at least in part, to macrophages, which the researchers showed more effectively killed LAC *in vitro* after savirin treatment. The bacterial clearance observed was similar to the phenotype of the *agr* deletion mutant without treatment. Another treatment with administration of 10 µg savirin delayed to 24 h and 48 h after initiation of infection led to a decreased reduction of ulcer area in the initial days of infection. Abscess area throughout treatment and ulcer area in the later days of infection did not change significantly relative to vehicle. Finally, they showed that exposing LAC to 5-µg doses of savirin in *in vivo* serial passaging through the skin of 10 individual mice did not lead to development of tolerance to savirin inhibition of *agr* function.

### OXA.

Waters et al. discovered and explored the ability of oxacillin (OXA), to which MRSA is resistant, to attenuate MRSA virulence when administered at sub-growth-inhibitory concentrations (Fig. 2C) (48). They rationalized that such dosing would increase MRSA methicillin resistance and subsequently downregulate the Agr system and virulence.

The group established sepsis in CD1 female mice via tail vain injection of 5 × 10^6^ CFU of a USA300 strain. The mice were sorted into groups of five, and the infection was given 9 h to establish before OXA was administered. One group of mice was treated with 75-mg/kg doses of OXA while another was treated with 7.5 mg/kg, both twice a day. Infection was allowed to proceed for 28 h or 1 week. On day 7, OXA treatment at both the low and high doses had resulted in significant reduction of bacterial burden, with kidney CFU reduced by 4 log units and blood CFU cleared completely (as observed from spleen). Additionally, while 8/10 untreated mice developed kidney abscesses, only 4/10 of the low-dose OXA mice and none of the high-dose OXA mice had visible kidney abscesses.

In a pneumonia model of MRSA infection, in which mice were intranasally infected with 2 × 10^8^ to 3 × 10^8^ CFU of a USA300 strain, the same treatments demonstrated positive results when given starting 3.5 h postinfection. Seven-day survival increased from 20% in the untreated group to 60% in both treatment groups. Additionally, the blood levels of the USA300 strain in the treated mice were 3 log units less than control. To confirm OXA-mediated attenuation of virulence, the researchers showed that OXA exposure represses cytotoxicity of USA300 culture supernatants on neutrophils. Transcriptome analysis further revealed how OXA may be attenuating virulence via modulation of gene expression. For example, repression of *agr* and activation of *rot* (repressor of toxins) were observed.

### PLNAs.

Da et al. took an antisense oligonucleotide approach to inhibiting the Agr system, choosing to target AgrA due to its being relatively conserved in all four *agr* allelic groups (49). Their oligonucleotide of choice was locked nucleic acid (LNA), a modified RNA nucleotide where the ribose moiety is modified with an extra bridge connecting the 2′ oxygen and 4′ carbon. Two antisense LNAs were synthesized and covalently conjugated with cell-penetrating peptide (KFF)3K, resulting in two peptide-conjugated locked nucleic acids (PLNAs), PLNA34 and PLNA522 (Fig. 2D). Both were shown to have no growth-inhibitory effect on LAC up to 40 µM. qRT-PCR showed that both PLNA34 and PLNA522 decreased *agrA* transcription at 12.5 μM, with PLNA34 producing the greater effect. Similar levels of decreased transcription were consequently observed for RNAIII. PLNA34 treatment was shown to suppress the expression of several *agr*-regulated toxin genes: *psm-α*, *psm-β*, *hla*, and *pvl*. Additionally, culture filtrates from PLNA34-treated cells had decreased hemolytic activity. They also exhibited a significantly reduced ability to lyse human neutrophils and induce chemotaxis in them. In C57BL/6J mice, 40 μM PLNA34 administered with subcutaneous injection of LAC resulted in the formation of smaller abscesses and abrogated the development of epidermal necrosis, ulceration, and dermal necrosis. These protective effects were accompanied on day 6 by a decrease in bacterial population of more than 4 orders of magnitude.

## PEPTIDE QUORUM QUENCHERS

The one peptide quorum quencher discussed here is a derivative of RNAIII-inhibiting peptide (RIP). RIP is endogenously produced by *S. aureus* and functions to inhibit quorum sensing.

### RIP derivative.

Simonetti et al. aimed to evaluate the *in vivo* efficacy of the combined administration of the tetrapeptide RIP derivative FS10 (Fig. 2E) and tigecycline in a mouse abscess model of MRSA infection (50). The methicillin-susceptible *S. aureus* (MSSA) strain ATCC 29213 and the MRSA strain ATCC 43300 were used throughout the study. Both strains showed susceptibility to tigecycline, which exhibited MICs of 0.12 and 0.25 µg/ml for the MSSA and MRSA strains, respectively. FS10, on the other hand, did not demonstrate growth inhibition at doses as high as 256 µg/ml. In terms of hemolytic activity, FS10 was highly ineffective at permeabilizing erythrocytes, causing <8% cellular disruption at high dose.

In the mouse wound infection model, infected BALB/c mice received treatment 24 h postinfection with either FS10 alone, tigecycline alone, or a combination. Tigecycline was given via intraperitoneal administration daily for 7 days at 7 mg/kg, while 20 µg FS10 was administered via wound dressing every 2 days. At day 8 postwounding, comparable trends were observed in MSSA and MRSA infections. As for the MRSA infections, the mean bacterial count in untreated controls was 4.5 × 10^9^ ± 1.4 × 10^9^ CFU/g of infected skin, which is significantly higher than the counts recovered from the treatment groups. FS10 alone had a slight growth-inhibitory effect, reducing bacterial count to 3.7 × 10^8^ ± 1.6 × 10^8^ CFU/g. Tigecycline alone had a strong effect, bringing the count down to 6.4 × 10^5^ ± 1.7 × 10^5^ CFU/g. The combination treatment led to the strongest growth-inhibitory effect, reducing bacterial count to 4.6 × 10^3^ ± 1.3 × 10^3^ CFU/g.

Finally, histological evaluation of the excised MRSA-infected tissues was done in order to assess the biological impact of different treatments on three wound healing parameters: epithelialization, granulation tissue, and collagen organization. A scoring system of 0 to 4 was utilized, with 0 being extensively disturbed and 4 being normal. Tigecycline treatment and FS10 treatment resulted in similar healing scores in the range of moderate healing across the three parameters (all scores between 2.80 and 3.00). The combination treatment, however, resulted in scores closer to that of the untreated control, all in the range of marked healing (scores between 3.20 and 3.50).

## NATURAL PRODUCT QUORUM QUENCHERS

Of the four natural product quorum quenchers discussed here, two are small molecules isolated from fungi while the other two are enriched plant extracts.

### OHM.

The group that reported on the synthetic small molecule savirin had previously pursued natural product QQs, identifying a number of polyhydroxyanthraquinones from the fungus *Penicillium restrictum* (51). A compound named ω-hydroxyemodin (OHM) (Fig. 2F) demonstrated the most potent inhibition of *agr* signaling in LAC and was therefore chosen for further study (52).

Daly et al. (52) first showed that OHM inhibits expression of all four *agr* types at concentrations that are not cytotoxic to LAC or to human alveolar (A549), kidney (HEK-293), and hepatocyte cell lines. They demonstrated this using strains expressing yellow fluorescent protein (YFP) under the control of the *agr*::P3 promoter. After ruling out AgrC as the target of OHM using an *agr* I isolate expressing constitutively active AgrC (R238H), they tested whether OHM inhibits *agr* function by targeting AgrA binding to promoter DNA (53). They found that OHM demonstrated a dose-dependent inhibition of AgrA_C_ binding to *agr* promoter DNA via molecular docking, an electrophoretic mobility shift assay, a bead-based assay of DNA-transcription factor binding, and surface plasmon resonance. OHM was found to also inhibit *agr* I expression in *S. epidermidis*, likely because the OHM binding site on AgrA_C_ is conserved between the species.

OHM was then assessed *in vivo* in a mouse model of MRSA skin infection (54). A load of 5 × 10^7^ to 7 × 10^7^ CFU of LAC was subcutaneously injected into mice with 5 µg OHM. By day 3, treated mice had abscess and ulcer areas less than half the size of control, abscess bacterial burden reduced by half a log unit, and markedly reduced weight loss. By day 7, bacterial burden was reduced by 1.5 log units. In the mice infected with LAC Δ*agr*, no differences were observed between the OHM- and vehicle-treated mice. These results demonstrate that OHM specifically disrupts *agr* signaling with no direct effects on the host. Bacterial clearance suggested an increased capacity of host immunity to combat infection in the absence of *agr* signaling and subsequent toxin production. Indeed, OHM treatment of LAC, but not LAC Δ*agr*, was found to increase *in vitro* bacterial clearance by both mouse macrophages and human polymorphonuclear leukocytes (PMNs). Additionally, treatment was found to approximately double PMN viability in a 2-h incubation with LAC but not LAC Δ*agr* supernatant, as indicated by LDH release.

### Ambuic acid.

Todd et al. explored the antivirulence potential of the fungal metabolite ambuic acid (Fig. 2G), rationalizing that it targets AgrB and thus AIP synthesis in *S. aureus* (55). They developed a USA300 strain with the *agr* locus deleted and the *agrBD* genes of the *agr* I type integrated at a phage attachment site, the genes under the regulation of the *sarA* P1 promoter. This mutant strain, therefore, not only produces AIP constitutively but would only see an inhibition of AIP production due to perturbations specific to signal biosynthesis. Mass spectrometric measurements were performed to quantify the AIP production of the wild-type and mutant strains upon treatment with ambuic acid and AIP-II, a known AgrC antagonist. Supporting ambuic acid’s role as an AIP synthesis inhibitor, AIP-II treatment resulted in inhibition of AIP production in the wild-type strain only, as expected, while ambuic acid treatment itself caused inhibition in both strains. Furthermore, ambuic acid treatment was found via Western blot analysis to inhibit MRSA production of alpha-toxin in a dose-dependent manner and was found via quantitative real-time PCR to inhibit RNAIII production, consistent with a repression of the Agr quorum sensing system.

A murine mouse model of MRSA skin infection was pursued to evaluate ambuic acid activity *in vivo*. Male BALB/c mice received 50-µl intradermal injections into the abdominal skin of inoculum suspensions containing 1 × 10^8^ CFU of LAC and either dimethyl sulfoxide (DMSO) or 5, 25, or 50 µg ambuic acid dissolved in DMSO. Over the course of the 14-day experiment, the single 25-µg prophylactic dose of ambuic acid was found to almost completely abrogate skin ulcer formation while the 5-µg dose roughly halved the lesion size. Additionally, ambuic acid treatment caused significantly less infection-induced morbidity in the mice than vehicle treatment, as assessed by weight loss. Infection by a LAC strain containing an *agr*-driven *lux* reporter revealed real-time suppression of *agr* activity *in vivo* by 5 µg ambuic acid over the course of a 5-h period postchallenge.

To confirm whether ambuic acid could potentially broadly target virulence for multiple Gram-positive pathogens, Todd et al. compared 50% inhibitory concentration (IC_50_) values for AIP synthesis inhibition in *Staphylococcus aureus* (*agr* I, II, and III), *Staphylococcus epidermidis* (*agr* I, II, and III), *Staphylococcus lugdunensis* (*agr* I), *Staphylococcus saprophyticus*, *Listeria monocytogenes*, and *Enterococcus faecalis*. Ambuic acid demonstrated IC_50_s of <25 µM in 8 of the 11 strains.

### *Castanea sativa* leaf extract.

Quave et al. reported on an enriched extract of *Castanea sativa* leaves, 224C-F2 (Fig. 2H), which demonstrated nonbactericidal QQ activity against LAC (39, 56). 224C-F2 exhibited inhibition of *agr* expression in all *agr* types with IC_50_s from 1.56 to 25 μg/ml. No effects on growth were observed at up to 100 μg/ml. QQ activity was confirmed by the ability of 224C-F2 doses as low as 0.25 μg/ml to significantly reduce delta-toxin production of the strain NRS385, a heavy exotoxin producer, as determined by high-performance liquid chromatography (HPLC). Additionally, doses as low as 6.25 μg/ml significantly reduced red blood cell lysis by LAC supernatants. 224C-F2 demonstrated lack of cytotoxicity *in vitro* to human keratinocytes, a panel of skin commensals, and murine skin. After 15 days of serial passaging in NRS385, 224C-F2 consistently reduced delta-toxin production, indicating no development of resistance to the QQ. 224C-F2 was then tested in an *in vivo* prophylactic model of MRSA infection.

A load of 1 × 10^8^ CFU of LAC (AH1263) or its *agr* deletion mutant (AH1292) with either 224C-F2 (5 μg or 50 μg) or DMSO was injected intradermally into abdominal skin of female C5BL/6 mice. Not only did 224C-F2 decrease infection-induced ulcer area in a dose-dependent manner, but it significantly attenuated infection-induced morbidity relative to vehicle (as assessed by weight loss). Specifically, a 5-μg dose reduced peak abscess area to one-third, while 50 μg almost completely prevented abscess formation throughout the 14-day experiment. Finally, mice receiving treatment alone did not display signs of dermal irritation or clinical illness.

### *Schinus terebinthifolia* berry extract.

Muhs et al. reported on an enriched *Schinus terebinthifolia* extract, 430D-F5 (Fig. 2I), with nonbactericidal QQ activity against all four MRSA (USA300) *agr* allelic groups (57). IC_50_s for inhibition of *agr* expression in the reporter strain assay were 2, 4, 4, and 32 µg/ml for *agr* I to IV, respectively. No growth inhibition was observed upon dosing with 64 µg/ml or less. 430D-F5 treatment caused significant, dose-dependent reduction in delta-toxin production in several *S. aureus* strains, alpha-toxin production in LAC, and LAC supernatant hemolytic activity *in vitro*. In assessing extract cytotoxicity, fluorescence microscopy and LDH release showed that treatment protected HaCaT cells from damage by NRS385 supernatants at concentrations as low as 8 µg/ml. Additionally, 430D-F5 exhibited limited potential for perturbing the skin microbiome, indicated by the high growth-inhibitory concentrations observed against a panel of common skin commensals. Intradermal treatment of mouse skin showed no deleterious effects.

In a prophylactic mouse model of MRSA skin infection, a single 50-μg dose of 430D-F5 administered with 1 × 10^8^ CFU of LAC almost completely attenuated skin ulcer formation. Treated animals also displayed significantly less morbidity relative to vehicle, as assessed by weight loss. A MRSA *agr*::P3 *lux* reporter confirmed that *agr* expression, and thus QS, was inhibited by the treatment.

## CONCLUSIONS

All studies utilized a mouse model of skin infection where the duration of treatment was at least about 1 week. Of the studies that determined bacterial count at the site of infection over time, most reported that the count was unaffected or slightly affected by around day 3 and then extensively reduced by around day 7. To address why this reduction might occur, the savirin and OHM studies presented evidence for both hits indicating that the reduction may be due at least in part to an increased capacity of the immune system to clear the infection. Related to this, while the AgrA-targeting F12 biaryl hydroxyketone greatly extended survival in a *Galleria mellonella* larva model of infection, in mice it yielded modest wound healing and was the only QQ hit covered in this review reported to cause no reduction of bacterial counts in mice. Such outcomes highlight the importance of tracking *agr* expression *in vivo* in a given infection model in order to confirm whether therapeutic effects coincide with real-time inhibition of *agr* expression. Another interesting outcome is that even though both savirin and OHM target AgrA_C_, only the former acts selectively against *S. aureus*. The binding sites of both QQ hits have been described via molecular docking. Savirin was docked to Tyr229 on *S. aureus* AgrA_C_, which in *S. epidermidis* is a Phe; OHM was docked near Arg218, a residue strictly conserved across staphylococcal species (58, 59). AgrB is yet another QS component successfully targeted by a QQ hit. Because of this and its conservation across many Gram-positive bacterial pathogens, AgrB represents an attractive target for targeted drug discovery efforts (60).

The contribution of the Agr system to MRSA skin infections is firmly established in the literature, whereas its role in other types of infection has received somewhat less attention. This, as well as the fact that the majority of MRSA cases are infections of the skin, contributes to the focus on QQ testing on *in vivo* models of skin infection. The question of whether QQ represents an effective therapeutic strategy for other types of infections must continue to be explored. In particular, some focus should be dedicated toward the actual QQ treatment, rather than prophylaxis, against various MRSA *in vivo* infection models. For instance, the current literature demonstrates that *agr* dysfunction is associated with MRSA bacteremia (61–65). Some studies suggest that the incidence of *agr* dysfunction in bacteremia is somehow beneficial, perhaps sustained by a need for cooperation between *agr*-positive cells and *agr*-negative cheaters in a systemic infection (66–68). Such studies call into questions the potential efficacy of QQ in bacteremia. On the other hand, it may be that QQ treatment induces large-scale shutdown of QS in the *agr*-positive segment of a bacteremia population, tipping the effective balance of *agr*-positive and *agr*-negative cells in the population. Such an outcome, which could perturb the ability of MRSA cells to cooperate effectively to sustain pathogenicity, cannot be ruled out by available studies. *In vivo* exploration of bacteremia and other forms of MRSA infection represents a powerful strategy for unraveling such uncertainties.

As MRSA QQs are emerging that demonstrate strong potential for drug development, the field will inevitably see exciting studies that explore the hits in more depth. While a priority is to explore curative QQ strategies against MRSA infection, the current progress on prophylaxis cannot be underestimated. For instance, MEDI4893 is a human anti-alpha-toxin monoclonal antibody developed by MedImmune that is currently in phase II clinical trials against *S. aureus* alpha-toxin in mechanically ventilated adult subjects for prophylactic use (69). While the success of MEDI4893 provides further validation of therapeutic use of targeting specific toxins, Agr-targeting QQs stand to target an entire repertoire of toxins and virulence mechanisms. Mechanistic studies will prove important as they will demonstrate whether different QQs affect virulence differently, providing valuable information for future drug development. There is much room and need for lead optimization in the discovery of QQs with improved bioactivity and pharmacokinetic properties such as serum half-lives that allow for less-frequent dosing. For instance, one strategy to optimize peptides is N-methylation, which has been shown both to restrict conformational flexibility, potentially increasing binding affinity to the target, and to yield resistance to hydrolysis by peptidases in the gut wall, allowing for improved oral bioavailability (70). QQ effects on commensals, especially growth inhibition, represent a key consideration for avoiding microbiome disruption and development of resistance mechanisms that could then be transferred to different bacterial species. Other side effects such as increased biofilm production, which has been associated with *agr* dysfunction, must also be considered. For instance, Quave et al. determined that 224C-F2 did not promote biofilm production and that this was likely due to its moderate biofilm-inhibiting activity (39). A QQ that exhibits no such activity may be helped *in vivo* with concurrent administration of a biofilm inhibitor. Moving forward, although *agr* I strains of *S. aureus* predominate in human disease, QQs that are further along in drug development should be tested in *in vivo* models of infection by strains besides LAC (4). Many other strains, such as MW2 (*agr* III), exhibit high virulence and are of great concern in the clinic.

A promising outlook is portrayed by the current literature on the *in vivo* efficacy of QQs for inhibiting MRSA virulence and pathogenicity. This is highlighted by the fact that, at this early stage in drug discovery, very favorable responses in animal infection models have been achieved. Additionally, outcomes of great therapeutic interest have been reported, such as the sensitization to obsolete antibiotics with the biaryl hydroxyketones and synergism with RIP derivative-antibiotic combination treatment. These preliminary data, however, must not be overanalyzed. For example, being at such an early stage in drug development, the QQ hits covered here likely have off-target effects yet to be explored. As with all other anti-infectives, resistance to QQs is likely to develop. Therefore, studies must be undertaken to elucidate potential rates of resistance. For most of the QQ hits discussed, resistance frequency studies have not yet been performed in which the bacteria are collected from treated animals and tested for return of virulence in the presence of the QQ. Given that these are not antibacterial agents but antivirulence agents, the development and spread of resistance may be relatively slow in theory. Resistance may be further slowed down by utilizing QQs as adjuvants to conventional antibiotics. Indeed, the study of synergism and resistance development in antibiotic-QQ combination therapy represents one of the most important and logical steps toward bringing QQs to the clinic.
